# Arthroscopic excision of heterotopic ossification in the rectus femoris muscle causing extra-articular anterior hip impingement

**DOI:** 10.1051/sicotj/2018036

**Published:** 2018-09-17

**Authors:** Naoki Nakano, Laughter Lisenda, Vikas Khanduja

**Affiliations:** Department of Trauma and Orthopaedics, Addenbrooke’s Hospital, Cambridge University Hospitals NHS Foundation Trust, Hills Road, Cambridge, CB2 0QQ UK

**Keywords:** Subspine impingement, Heterotopic ossification, Extra-articular hip impingement, Arthroscopy

## Abstract

Subspine impingement is an extra-articular hip impingement syndrome that usually occurs when there is abnormal contact between an enlarged or malorientated anterior inferior iliac spine (AIIS) and the distal anterior femoral neck in straight flexion of the hip. We present the case of a 13-year-old boy with a history of left groin pain and loss of range of movement of the hip for over six months following an avulsion fracture of the AIIS during a game of rugby. He was diagnosed with subspine impingement secondary to a large lesion of heterotopic ossification in the rectus femoris; this was dissected and extracted from the muscle in toto arthroscopically. This case highlights the importance of heterotopic ossification after injury as an important cause for subspine impingement in the young adult hip. This is the first report and describes subspine impingement secondary to a large lesion of heterotopic ossification.

## Introduction

Groin pain is a common symptom in individuals involved in high-level sports, with pain arising from an intra- or an extra-articular source [1–3]. It is well known that femoroacetabular impingement (FAI) is an important intra-articular cause of pain and loss of movement in the young, non-arthritic patient [3–9]. Recently, extra-articular impingement of the hip, which is caused by abnormal contact between the extra-articular regions of the proximal femur and pelvis and may coexist with intra-articular FAI, has been described [10,11]. This is also an important contributor to the differential diagnoses of pain and mechanical symptoms in and around the hip in the young adult [10,11].

Subspine impingement is an extra-articular impingement syndrome and the patho-anatomy of this pathology was first reported in 2008 [1]. The syndrome was described in a 30-year-old male who had a hypertrophic anterior inferior iliac spine (AIIS), leading to symptomatic impingement at the head-neck junction. The AIIS is located just above the anterosuperior portion of the acetabular rim and is the origin of the straight head of the rectus femoris tendon. Subspine impingement can also occur because of an avulsion injury of the AIIS due to the excessive muscular activity of the rectus femoris. Rapid high-energy knee flexion combined with hip extension has been proposed as a possible pathological mechanism for this condition [12].

In this report, we present a rare mechanism for the causation of subspine impingement in a young boy occurring secondary to the formation of a large lesion of heterotopic ossification following an avulsion fracture of the AIIS which was managed arthroscopically.

The patients and their families were informed that data from the case would be submitted for publication, and gave their consent.

## Case presentation

A 13-year-old boy was referred to our tertiary young adult hip service by a Paediatric Orthopaedic Surgeon for evaluation of left groin pain and a decreased range of movement in the left hip. He was a keen rugby player and had experienced an avulsion fracture of the AIIS during a rugby game which was played without a warm up six months previously. MRI just after injury showed a single bony fragment measuring 12 mm × 4 mm × 12 mm at the rectus femoris origin of the AIIS and it was retracted inferiorly 3 cm, anteriorly 1 cm and laterally 0.5 cm. Conservative treatment was advised by the Paediatric Orthopaedic Surgeon and he was referred to a physiotherapist for mobilisation and subsequently muscle strengthening.

**Figure 1:**
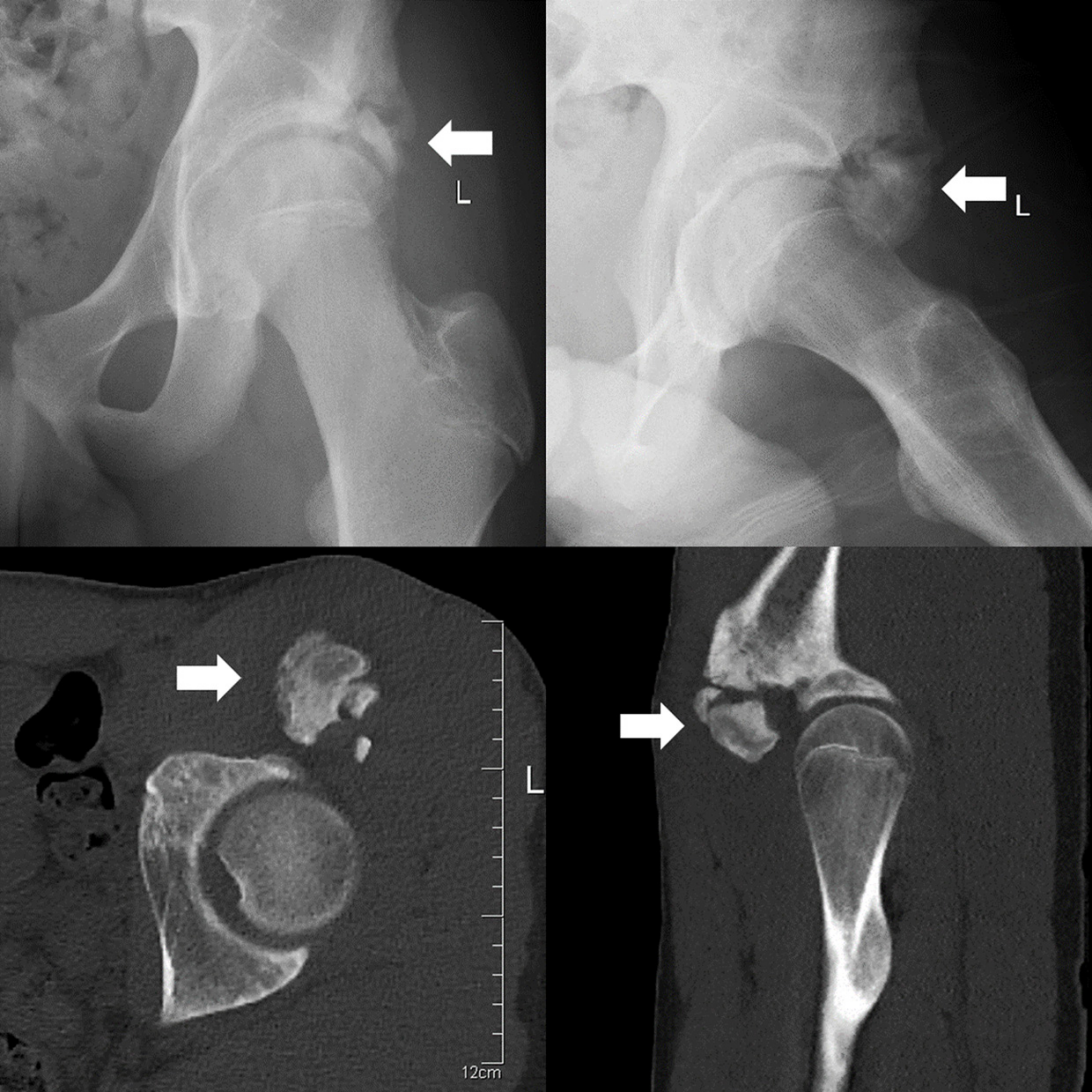
Pre-operative radiograph and CT scan. Arrows show the area of heterotopic ossification that occurred in the rectus femoris muscle near the origin of the anterior inferior iliac spine.

Five months post-injury he was progressing well with the physiotherapy, although, he still felt a sharp pain in his left groin when running with a ball while playing rugby. Flexion was restricted to 70° and a decrease in internal rotation in comparison with the opposite side was observed while abduction, adduction, extension and external rotation were comparable to the other side. There was no significant tenderness and no bruise in the region of the groin, and no distal neurovascular deficit. Plain radiographs demonstrated hypertrophic calcification in the region of the rectus femoris avulsion, whilst the hip joints were normal in appearance. A CT scan showed well corticated heterotopic bone formation at the site, measuring approximately 3.5 cm at maximum diameter (Figure 1). Motion analysis using the three-dimensional CT datasets clearly showed the impinging area especially in 70° of hip flexion.

**Figure 2:**
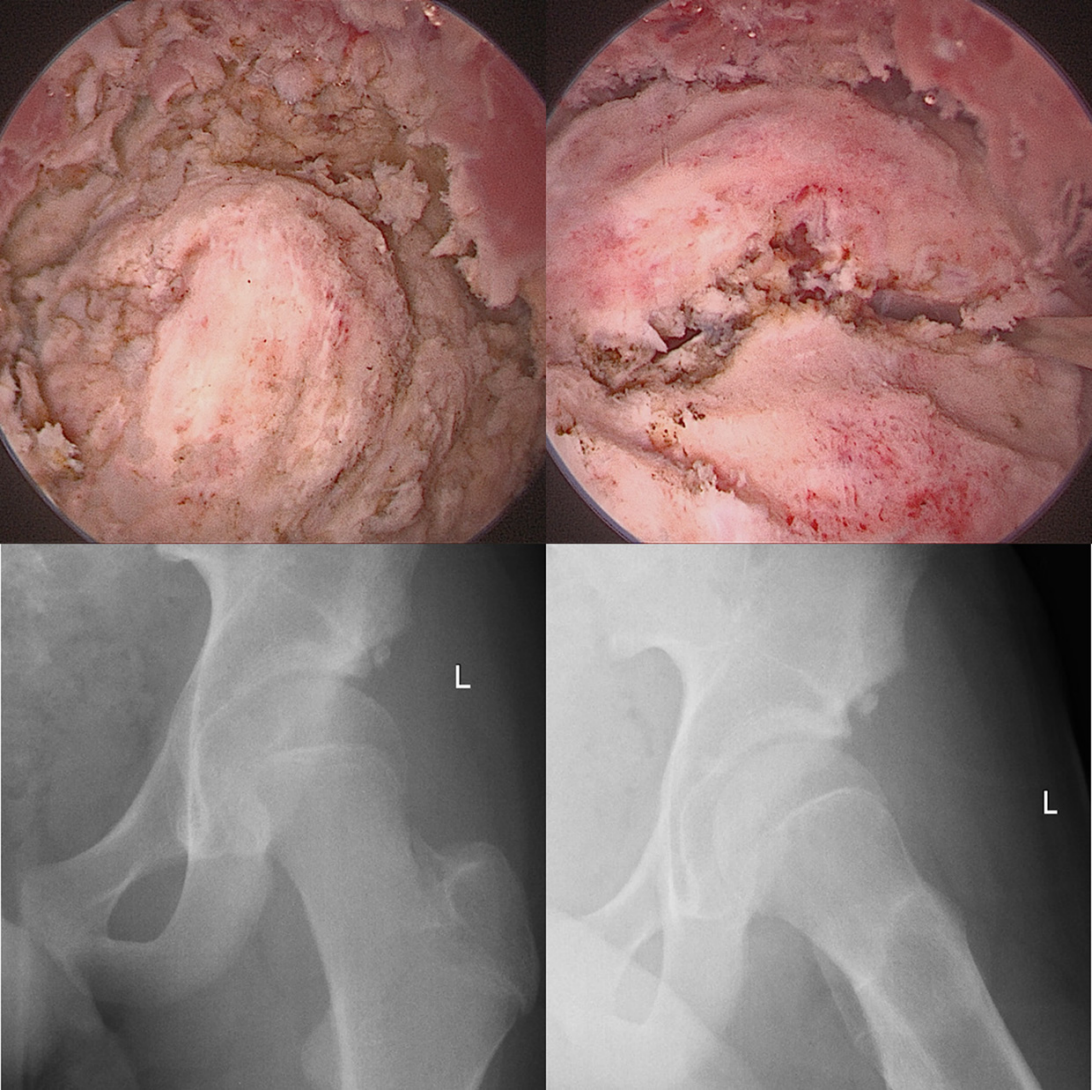
Arthroscopic images of the heterotopic ossification that occurred in the rectus femoris muscle which was excised and extracted via an incision made on the anterolateral aspect of the thigh, and post-operative radiograph which shows that the heterotopic ossification lesion was cleared completely.

Both conservative and surgical management were suggested and discussed with the patient and his parents. They chose to go ahead with surgery, as he could not play rugby well because of the pain and restricted range of movement. The patient wanted to play rugby at a fairly high level, hopefully nationally, in the future. At arthroscopy, he was found to have a well corticated large lesion of heterotopic ossification, and it was dissected carefully from the muscles (Figure 2). Once the dissection was completed, it was extracted via an incision to deliver the lesion. A dynamic impingement test was performed on the table to ensure there was no residual impingement and the impingement lesion was resected with a 5.5 mm arthroscopic burr. A thorough washout was carried out before closure.

The patient was advised not to perform any rotational activities in deep flexion for six weeks postoperatively and to follow the 16-week post-operative rehabilitation protocol. He was also advised prophylaxis against heterotopic ossification. Eight weeks following the procedure, the wound had healed well and there were no obvious signs of complications such as infection or deep vein thrombosis. Along with this, he demonstrated a pain-free, fully functional range of movement in his left hip joint and was delighted with his progress. Plain radiographs showed no further signs of calcification (Figure 2). He was advised to continue to attend physiotherapy for at least another eight weeks and to engage in more gentle sporting activities like cricket and basketball, which he had enjoyed before the injury. At the 1-year follow-up, he remains asymptomatic with a full range of movement in his hip and continues to play rugby at a high level.

## Discussion

We have described a large heterotopic ossification after an avulsion fracture of the AIIS which caused subspine impingement in a 13-year-old boy. It was dissected from muscles arthroscopically and extracted. There has been no other report describing subspine impingement secondary to a large heterotopic ossification thus far.

Patients with subspine impingement usually present with anterior hip or groin pain on flexion and internal rotation as well as mechanical symptoms including a loss of range of movement, sometimes associated with a grinding sensation [13]. In adolescents, repetitive traction injuries can cause apophysitis, hypertrophy, inferior displacement of the apophysis or fracture, which can lead to malunion resulting in an enlarged AIIS or a bony protrusion [12]. An avulsion fracture of the AIIS can normally be detected immediately after the injury on a plain radiograph, but subtler findings are often missed [14,15]. To diagnose this condition more precisely from a biomechanical view, it is beneficial to use three-dimensional CT datasets which make pre-operative dynamic simulations (motion analysis) possible, in order to detect a direct osseous impingement between the AIIS deformity and the femoral neck at maximum hip flexion. In the current case, motion analysis clearly showed the impingement area at 70° of hip flexion, which was identical to the patient’s maximum flexion angle.

Hetsroni et al. [16] published a classification of AIIS morphology based on the distance between AIIS and the anterosuperior acetabular rim. Three types of morphology were established: Type I when there was a smooth ilium wall between the AIIS and the acetabular rim; Type II when the AIIS extended to the level of the rim; and Type III when the AIIS extended distally to the acetabular rim. They reported that Type II and III variants are associated with a decrease in hip flexion and internal rotation, supporting the rationale for considering AIIS decompression for variants that extend to and below the rim. In this case, the heterotopic ossification lesion was large in size, and pathomorphology and treatment were in accordance with those in Type III. Arthroscopic resection of the AIIS for subspine impingement is thought of as a relatively safe procedure, although a case of avulsion of the rectus femoris direct head following revision hip arthroscopy for the treatment of subspine impingement in a 23-year-old professional footballer was reported recently [17].

It is difficult to predict or prevent the formation of heterotopic ossification; prophylaxis with Indomethacin or naproxen may be considered for four to six weeks after injury or arthroscopy, and patients should be followed up (range of movement and radiograph) for up to 12 months if heterotopic ossification does appear on the first plain radiograph [18]. Also, as it is theorised that bone debris generated during osteoplasty in hip arthroscopy might trigger the formation of new bone, prevention of heterotopic ossification requires the hip joint to be lavaged carefully at the end of the procedure to ensure that all bony debris from the osteoplasty has been cleared [14].

Surgeons involved in the care of young adults with hip pain should keep subspine impingement secondary to the heterotopic ossification in mind as a differential, especially in a patient with groin pain and mechanical symptoms after injury.

## Conflict of interest

The authors declare that they have no conflicts of interest in relation to this article.
